# Evaluation of adhesion strength, corrosion, and biological properties of the MWCNT/TiO_2_ coating intended for medical applications

**DOI:** 10.1039/d3ra05331h

**Published:** 2023-10-16

**Authors:** Dorota Rogala-Wielgus, Beata Majkowska-Marzec, Andrzej Zieliński, Katarzyna Roszek, Malwina Liszewska

**Affiliations:** a Division of Biomaterials Technology, Institute of Manufacturing and Materials Technology, Faculty of Mechanical Engineering and Ship Technology, Gdansk University of Technology 11 Narutowicza Str. 80-233 Gdańsk Poland dorota.wielgus@pg.edu.pl; b Faculty of Biological and Veterinary Sciences, Nicolaus Copernicus University in Toruń Lwowska 1 Str. 87-100 Toruń Poland; c Institute of Optoelectronics, Military University of Technology Kaliskiego 2 Str. 00-908 Warsaw Poland

## Abstract

Multi-wall carbon nanotube (MWCNT) coatings are gaining increasing interest because of their special properties used in many science fields. The titania coatings are known for their improvement of osteoblast adhesion, thus changing the surface architecture. Bi-layer coatings comprising 0.25 wt% of the MWCNTs and 0.30 wt% of titania (anatase structure) were synthesized in a two-stage procedure using the electrophoretic deposition method (EPD). The MWCNT and TiO_2_ coatings were deposited with voltage and time parameters, respectively, of 20 V and 0.5 min, and 50 V and 4 min. EDS, AFM, SEM, Raman spectroscopy, nano-scratch test, potentiodynamic corrosion tests, wettability studies, and cytotoxicity determined with MTT (3-(4,5-dimethylthiazol-2-yl)-2,5-diphenyltetrazolium bromide) test on human dermal fibroblasts (HDF) and mouse osteoblast precursors (MC3T3), and lactate dehydrogenase (LDH) activity test were carried out on examined surfaces. The prepared MWCNT/TiO_2_ coating is uniformly distributed by MWCNTs and agglomerated by TiO_2_ particles of size ranging from 0.1 to 3 μm. Raman spectroscopy confirmed the anatase structure of the TiO_2_ addition and showed typical peaks of the MWCNTs. The MWCNT/TiO_2_ coating had higher roughness, higher adhesion strength, and improved corrosion resistance compared to the MWCNT basic coating. The results of biological tests proved that physicochemical properties of the surface, such as high porosity and wettability of MWCNT/TiO_2_-coated material, would support cell adhesion, but toxic species could be released to the culture medium, thus resulting in a decrease in proliferation.

## Abstract 2

Herein a MWCNT/TiO_2_ coating was shown to have higher roughness, adhesion strength and corrosion resistance compared to MWCNT, also with high porosity and wettability to support cell adhesion. However, the release of toxic species results in decreased proliferation.

## Graphical abstract

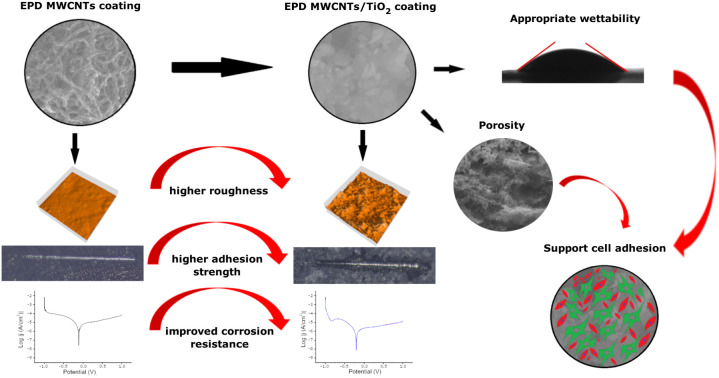

## 1 Introduction

Among nanomaterials, carbon allotropes of carbon nanotubes (CNTs) have gained significant attention owing to their unique properties: chemical inertness, exceptionally high mechanical strength, significant electrical conductivity, and often content-related optical properties. Therefore, construction and functional materials containing CNTs have been developed in various fields of interest. The last reviews on such materials have shown the great potential of CNTs, in medicine as reinforced polymer-based composites,^1^ in tissue engineering,^2^ and as optical contrast agents for cell imaging,^3^ in electronics for the construction of CNT-based nanogenerators,^4^ supercapacitors, and high-performance batteries,^5^ in industry for the development of construction reinforced polymers^6^ and reinforcement of cement concrete,^7,8^ and in chemistry for adsorptive removal of metal ions,^9^ and as catalysts in hydrogenation processes.^10^

CNTs have seldom been proposed as coating components and exceptionally as single layers. Recently, such implementations have resulted in abrasion-resistant, photothermal, and superhydrophobic anti-icing coatings,^11–14^ anticorrosion and mechanically resistant coatings,^15–18^ antifouling coating,^19^ and coatings designed especially for heat exchangers and microwaves.^20,21^

CNT-based coatings deposited on titanium and its alloys, including NiTi, are infrequent and highly diversified. The carboxylic multi-wall carbon nanotube (MWCNT) coating on Ti alloy with CNT content from 0.05 to 0.2 wt% was designed to decrease friction and wear rates.^14^ The most popular ones are hydroxyapatite (HAp) coatings reinforced with CNTs in amounts of up to 2 wt%^22^ or 0.01 to 0.1 wt%,^23^ which are expected to increase hardness and adhesion strength. The HAp–Ti coatings doped with 1 wt% of MWCNTs on NiTi alloy^24^ and (Ce, Sr)HAp–agar–chitosan–MWCNTs (an amount unknown) on Ti^25^ were proposed for their better hardness, adhesion, and biological behavior. The tantalum oxide and CNTs were obtained by applying the sol–gel method to increase corrosion resistance, adhesion, and bioactivity.^26,27^ MWCNTs deposited on Ti in amounts of 5, 10, and 20 μg cm^−2^ were planned to enhance osseointegration.^28^ All these coatings were specially designed and investigated as potential candidates for surface modifications of titanium implants. Moreover, the self-lubricating coatings Al3Ti–3CNTs–3Cu–7SiC were produced by laser melting for light material processing.^29^ The CNT–polysiloxane coating was proposed as chemically resistant, durable, and highly hydrophobic,^30^ and epoxy resin vapor-deposited with CNT coatings for aircraft applications^31^ was developed.

The coatings are prepared by employing different techniques. They include mostly plasma spraying,^32–34^ electrophoretic deposition method (EPD),^35–37^ laser cladding,^38^ and electrostatic spraying.^39^ However, electrophoretic deposition (EPD) is also widely used to prepare CNT coatings^40,41^ because it enables the manipulation of the CNTs to deposit on surfaces with variable shapes, including flat or balk, gives control over coating parameters, is low-cost and time-saving.^42^

The addition of CNTs to the coatings deposited on titanium and its alloys can improve several properties important for implants. However, CNTs can form agglomerates because of the significant van der Waals chemical force, which adversely affects the properties of the coatings. The proposed techniques include covalent or non-covalent functionalization in a liquid rather than in a solid.^43^ Recently, modification of MWNCT surfaces by ZrO_2_ nanoparticles was shown to enhance the dispersion of MWCNTs and increase their adhesion to the epoxy matrix and its mechanical and fracture behavior.^44^ Among them are mechanical properties enhanced by CNTs present as the adhesion strength from 18.5 to 24.2 MPa at 1 wt%,^22^ from 17.2 MPa to 32.1 MPa at 1 wt%,^24^ from 21 to 29 MPa,^25^ and from 17.5 to 32.1 MPa^37^ and observed only qualitatively by the homogenous dispersion of CNTs in the matrix for CNT content in the range of 2–6 wt%.^34^ The hardness was elevated from 6.12 to 7.22 GPa at 2 wt%^22^ and from 72 HV to 405 HV at 1 wt%.^24^ Young's modulus increased from 115 to 135 MPa at 2 wt%,^22^ and also from 70 to 400 MPa.^37^ The deposition of MWNCTs on titanium caused about a 10% decrease under dry conditions, but even a 90% decrease in SBF (simulated body fluid),^14^ and a moderate change in wear volume from 14.14 × 10^6^ to 10.6 × 10^6^ mm^3^ was found after adding CNTs to TiO_2_ in the sprayed-made coating.^32^ A decreasing current density was noticed for the CNT–Hap coating from 0.54 to 0.05 μA cm^−2^ at 1 wt% CNTs.^22^ On the contrary, after adding MWCNTs to HAp–Ta_2_O coating, corrosion current density increased from 0.011 to 0.021 μA cm^−2^.^27^ The contact angle changed from 40° for Ti to 31° for Ti–Ta_2_O_5_.^27^

Biological behavior has often been reported to be positively affected by the addition of CNTs. The coating composed of tantalum oxide and CNTs modified with phosphonic acid^26,27^ improved bioactivity, and the titanium substrate electrochemically anodized and coated with CNTs increased the proliferation of MC3T3 cells and induced the formation of hydroxyapatite, making the surface proper for application in dental implants.^45^ The HAp–Ti–MWCNT composite coating was claimed to improve cellular proliferation and growth on the surface of NiTi alloy.^37^ For Ti and its alloys as substrates, in hydroxyapatite coatings, the addition of CNTs increased cell viability.^22^ In more complex carrageenan–chitosan–(Ce, Sr)HAp coatings, the MWCNTs also positively affected cell adsorption in *in vivo* tests,^25^ for collagen–CNTs, enhanced cell proliferation was observed,^28^ and for HAp–SCNTs, improved bioactivity determined by MTT and ALP essays, cell morphology and proliferation appeared.^23^

The basic mechanical properties of the multi-wall carbon nanotube coating with titania (MWCNT/TiO_2_), which is the object of this research, were examined and discussed in our previous work.^36^ Herein, the presented results include adhesion strength, corrosion resistance, and biological behavior, which together strongly justify the potential application of the developed coating for implantology, particularly endoprosthesis and dental implants. The literature shows that a mixture of TiO_2_ polymorphs at a concentration of 80% in anatase and 20% in rutile is the most effective in biomedical applications.^46,47^ Thus, TiO_2_ in crystalline anatase form is more active than TiO_2_ in rutile form, and at the same time, it is more effective for antimicrobial purposes.^47^ However, the anatase TiO_2_ polymorph shows worse corrosion resistance^48^ and cytotoxicity properties^47^ compared to the rutile form. In this study, we used the anatase; it has a large Young's modulus of 177.24 GPa^49^ and a small share modulus of 42.69 GPa,^49^ which are properties demanded by coatings intended for the endoprosthesis.

## 2 Experimental

### 2.1 Preparation of the substrate surface

The round-shaped specimens of 20 mm diameter and 4 mm thick were prepared from Ti13Nb13Zr alloy (Xi'an SATE Metal Materials Development Co., Ltd., Xi'an, China) of the following composition: 13.18 wt% Nb, 13.49 wt% Zr, 0.085 wt% Fe, 0.035 wt% C, 0.004 wt% H, 0.078 wt% O, <0.001 wt% S, 0.055 wt% Hf and remaining Ti. The surface preparation was further described in ref. 35 and included surface grounding with SiC paper of up to #800 grit; cleaning in acetone (Chempur, Piekary Śląskie, Poland) for 2 min; distilled water for 2 min; etching in 5% solution of hydrofluoric acid (Chempur, Piekary Śląskie, Poland); and rinsing in distilled water. This prepared substrate was assigned an MR value (native material).

### 2.2 Preparation of CNT coatings

The MWCNT (–COOH modified, 3D-Nano, Krakow, Poland) coatings were prepared using EPD with parameters and bath composition, as shown in Table 1.

**Table 1:** EPD process parameters for the examined coatings

Material	Substrate	Deposited materials	Content of component in a bath (wt%)	EPD time (min)	EPD voltage (V)
MR	Ti13Nb13Zr	—	—	—	—
MWCNTs^36^	Ti13Nb13Zr	MWCNTs	0.25	0.5	20
MWCNT/TiO_2_ ^36^	Ti13Nb13Zr	(I) MWCNTs	0.25	0.5	20
		(II) TiO_2_	0.30	4	50

The MWCNT EPD bath was composed of 0.25 wt% of MWCNTs suspended in distilled water. Before the deposition process, the suspension was ultrasonically dispersed for 1 h in an ultrasonic bath (MKD-8, MKD Ultrasonic, Warsaw, Poland), with power and frequency of 300 W and 25 kHz, respectively. The MWCNT/TiO_2_ coating was prepared in a two-stage process. First, the MWCNT layer was formed by EPD in the bath of the composition described above. Then, the second, TiO_2_ (3D-Nano, Krakow, Poland) layer was created by EPD in the bath comprising 0.30 wt% of TiO_2_, isopropyl alcohol as a solvent, and 1 wt% of polysorbate 20 (Tween 20, Sigma-Aldrich, Poznan, Poland). The suspension was then ultrasonically dispersed for 6 h, and the power and frequency were set the same as those observed during the dispersion of the MWCNT suspension.

The EPD for the MWCNT coating was conducted with Ti13Nb13Zr as a positive electrode and stainless steel as a negative electrode, while for the MWCNT/TiO_2_ coating, the electrodes were converted. The distance between the electrodes was about 0.5 cm.

### 2.3 Chemical composition and topography

The surface topography was evaluated using an atomic force microscope (AFM NaniteAFM, Nanosurf, Bracknell, Great Britain) in non-contact mode at 20 mN force. The average roughness index *S*_a_ values were estimated based on 512 lines made in the area of 80.4 × 80.4 μm.

A high-resolution scanning electron microscope (SEM JEOL JSM-7800F, Tokyo, Japan) with an LED detector was used at a 5 kV acceleration voltage to observe the surface morphology.

An X-ray energy dispersive spectrometer (EDS) (Octane Elite 25, EDAX Ametek, Berwyn, PA, USA) was used to evaluate the chemical composition of the MWCNT/TiO_2_ coating.

### 2.4 Chemical structure and crystallography

The measurements were carried out using a Raman microscope (Renishaw InVia Plc., Wotton-under-Edge, UK) equipped with an EMCCD detector (Andor Technology Ltd., Oxford Instruments, Belfast, UK) and the objective lens set at 20×. The wavelength of the laser radiation during Raman spectroscopy tests was 532 nm; the measurement time, the measurement counts at significant points, and the laser radiation power of the MWCNT sample were 1 s, 5, and *ca.* 0.2 mW, respectively, and those of the MWCNT/TiO_2_ coating were 0.5 s, 10, and *ca.* 1 mW, respectively. Raman spectra measurements were prepared as maps consisting of the mean values of 100 points and the standard deviation of the signal sample. The collected Raman spectra were processed in WiRE 5.5 software and then averaged using CasaXPS software.

### 2.5 Adhesion determination

Nano-scratch tests were carried out using the NanoTest™ Vantage (Micro Materials, Wrexham, Great Britain) in increasing load mode from 0 to 200 mN at a distance of 500 μm, with a loading rate of 1.3 mN s^−1^. The adhesion strength was evaluated based on the critical load (*L*_c_), which was determined by the abrupt change in critical friction (*F*_t_) in the *F*_t_ to critical force (*F*_n_) relation graph. In the end, the scratches were investigated using a light microscope (BX51, OLYMPUS, Tokyo, Japan), and the results were shown as mean ± SD (*n* = 5).

### 2.6 Corrosion behavior

Corrosion tests were carried out using a potentiostat (Atlas 0531, Atlas Sollich, Gdańsk, Poland), with the AtlasCorr05 software, by calculating the corrosion potential (*E*_corr_) and corrosion current density (*j*_corr_) based on Tafel extrapolation. The sample served as the working electrode, a platinum rod as the counter electrode, and a saturated calomel electrode as the reference electrode, immersed in Ringer's solution (composition: NaCl, 8.6; CaCl_2_, 0.33; KCl, 0.30 g L^−1^) at a temperature of 37 °C. The open circuit potential (OCP) was stabilized for 1 h, and potentiodynamic measurements were performed from −1.0 V to 1.0 V at a scan rate of 1 mV s^−1^.

### 2.7 Wettability

The water contact angle (CA) was evaluated using a goniometer (Contact Angle Goniometer, Zeiss, Oberkochen, Germany) with the pendant drop mode. Wettability was measured for 10 s after the drop fell down the surface, and the CA result for each surface, shown as a mean ± SD (*n* = 3), was red after 5 s.

### 2.8 Biological characterization

Cytotoxicity studies were conducted using a human dermal fibroblast (HDF, Biokom, Poland) cell line and mouse osteoblast precursors (MC3T3, Sigma-Aldrich, Germany). The HDF cells were grown in DMEM-LG (Dulbecco's Modified Eagle's Medium, Low Glucose) and MC3T3 in EMEM (Eagle's Minimum Essential Medium), both supplemented with 10% Fetal Bovine Serum (FBS) according to ref. 50. Before the experiment, the approximately 1 × 10^4^ cells in culture media of 5 μL were seeded on the tested materials, different specimens in separate wells of a 12-well plate, and left for 3 h for adhesion. Then, the culture medium was added and incubated for 24 h and 72 h in a direct test. In an indirect test, the examined specimens were immersed in culture media for 72 h, and then as prepared suspension was used to HDF and MC3T3 cell culture seeded 24 h before in a 12-well plate. Cell viability was assessed based on an MTT (3-(4,5-dimethylthiazol-2-yl)-2,5-diphenyltetrazolium bromide) (Sigma-Aldrich, Germany) assay, which showed cell ability to reduce MTT. The absorbance of the reduced formazan was measured at 570 nm using a Synergy HT Multi-detection reader (BioTek Instruments, Winooski, VT, USA).

To check the integrity of the cell membranes, a lactate dehydrogenase (LDH) activity test was performed. The decrease in NADH (nicotinamide adenine dinucleotide, reduced disodium salt) (Sigma-Aldrich, Germany), indicating an increase in the number of damaged cells in the culture medium, was measured after 24 h and 72 h. To 150 μL of culture media, 25 μL of NADH (2.5 mg mL^−1^) and 25 μL of sodium pyruvate (2.5 mg mL^−1^) (Sigma-Aldrich, Germany) were added. The LDH activity was assessed spectrophotometrically (Synergy HT Multi-detection Reader, BioTek Instruments, Winooski, VT, USA) by measuring absorbance at 340 nm. The result for each sample was shown as a percentage of positive control samples treated with 1% Triton X-100 and labeled as 100% damaged cells.

### 2.9 Statistical data

The experimental values were provided as mean ± standard deviation (SD), and the statistical significance of differences between each specimen was evaluated utilizing Origin 8 by one-way analysis of variance (ANOVA), as depicted in figures (*).

## 3 Results and discussion

### 3.1 Chemical composition and topography

**Fig. 1:**
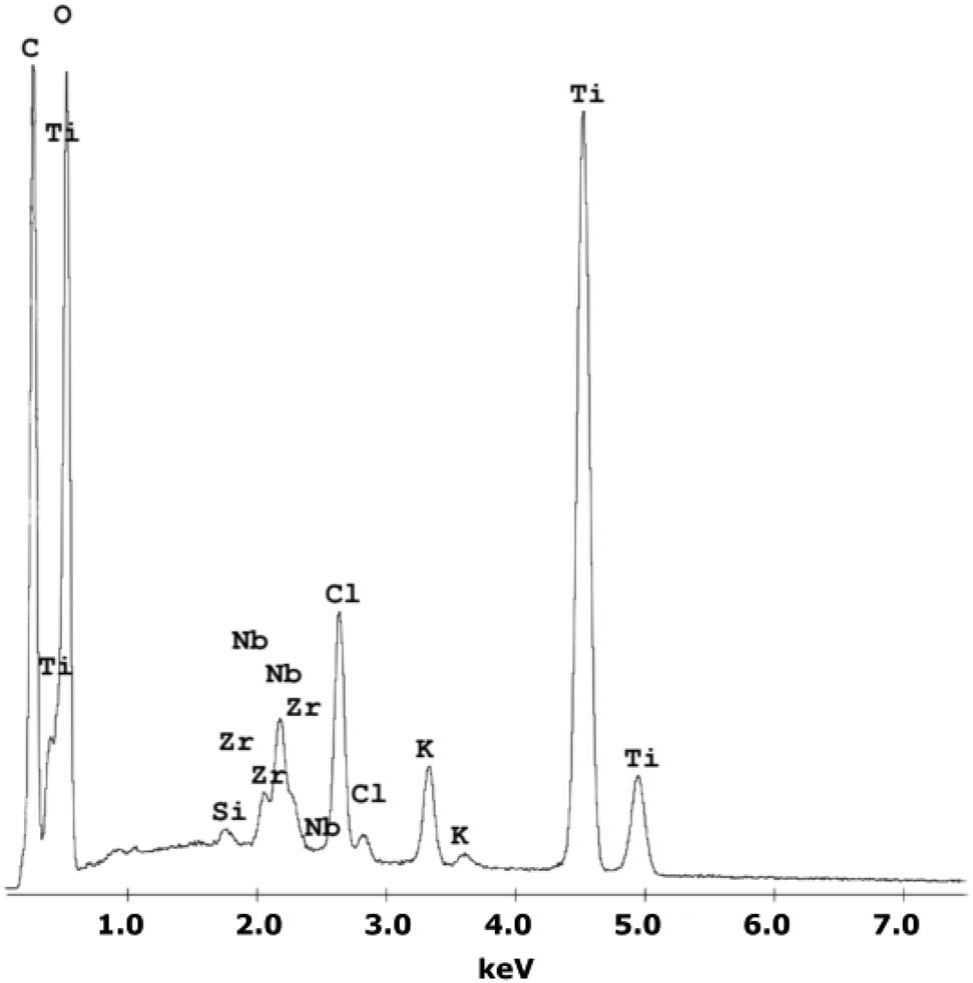
The EDS spectra of the MWCNT/TiO_2_ coating.

A chemical EDS-based analysis of the MWCNT/TiO_2_ coating, shown in Fig. 1, was conducted to detect the elements present in the coating. Titanium, zirconium, and niobium are the elements that originate from the substrate material, and carbon, titanium, and oxygen originate from the coating. Additionally, some elements, Cl and K, are impurities that appear in the deposition process. The presence of high peaks of carbon and titanium proves the deposition of the MWCNT/TiO_2_ layer.

Table 2 shows the surface roughness *S*_a_ values for all examined surfaces estimated with an experimental error of less than 0.05 μm.

**Table 2:** Roughness *S*_a_ parameter for the examined surfaces

Material	Roughness *S*_a_ parameter (μm)
MR	0.069^a^
MWCNTs	0.159^a^
MWCNT/TiO_2_	0.901

a Results are shown before in ref. 64.

**Fig. 2:**
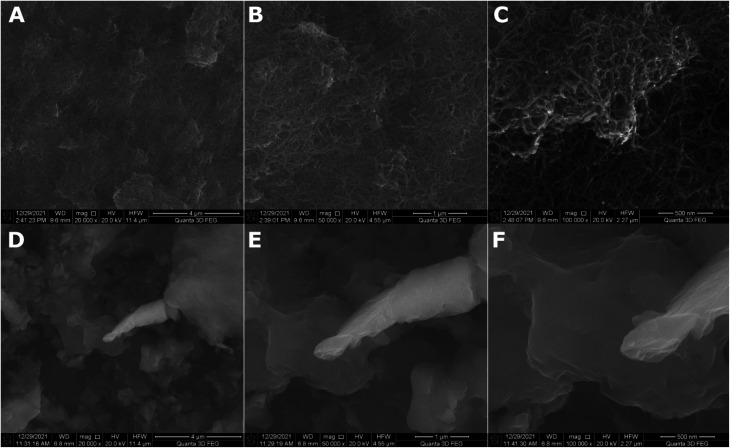
SEM surface topography of the MWCNT coating (A–C) and the MWCNT/TiO_2_ coating (D–F) demonstrated in different resolutions.

The roughness of the MWCNT coating increased more than 5.5-fold with the addition of titania nanoparticles. A similar effect was observed in our previous work.^36^ This is caused by the small TiO_2_ nanoparticles agglomerating on the MWCNT surface, as observed in Fig. 2, showing the SEM topography of the MWCNT and MWCNT/TiO_2_ coatings.

The MWCNT coating (Fig. 2A–C) is uniformly distributed. The difference in thickness in particular areas of the coatings was previously estimated at 0.55 μm.^36^Fig. 2D–F demonstrates the SEM topography of the MWCNT/TiO_2_ coating. The MWCNT coating is transparent and thoroughly covered by titania agglomerates of different shapes and sizes ranging from 0.1 to 3 μm. In our previous study, the average TiO_2_ aggregate surface area was approximately 1.5 μm^2^.^36^ The lamellar structure of each agglomerate can be observed, probably resulting from the coating's synthesis method, where coatings are built layer by layer during the EPD deposition process. The coating thickness measured earlier was 2.016 μm.^36^

### 3.2 Chemical structure and crystallography

**Fig. 3:**
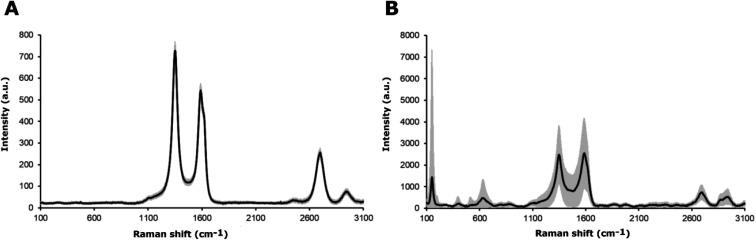
Raman spectra of (A) the MWCNT coating and (B) the MWCNT/TiO_2_ coating, where the black line presents an average Raman spectrum and the gray area is a standard deviation of the signal.

The chemical characterization of the coatings was based on Raman spectroscopy. The Raman spectra for the MWCNT and the MWCNT/TiO_2_ coating are shown in Fig. 3. The values of the bands, with appropriate assignments for the characteristics of the bands, are listed in Tables 3 and 4, for MWCNT and MWCNT/TiO_2_ coatings, respectively.

**Table 3:** Raman active optical phonon modes for the MWCNT coating compared with MWCNT powder and literature results for MWCNT coating

	D band (cm^−1^)	G band (cm^−1^)	G′ band (2D band) (cm^−1^)	G′ band (2D band) (cm^−1^)	Ref.
MWCNTs	1350	1588	2699	2942	—
MWCNT coating on Ti13Nb13Zr substrate	1345	1580	2690	—	61
MWCNT powder	1346	1583	2694	—	62
MWCNT composite (prepared by evaporation and drying process)	1340	1574	2694	—	51

**Table 4:** Raman active optical phonon modes for the MWCNT/TiO_2_ coating compared with rutile and anatase TiO_2_ structures

	E_g(1)_ (cm^−1^)	E_g(2)_ (cm^−1^)	B_1g(1)_ (cm^−1^)	A_1g(1)_ + B_1g(2)_ (cm^−1^)	E_g(3)_ (cm^−1^)	D band (cm^−1^)	G band (cm^−1^)	G′ band (2D band) (cm^−1^)	G′ band (2D band) (cm^−1^)	Ref.
MWCNT/TiO_2_	150	200	401	519	630	1348	1587	2687	2938	—
MWCNT/TiO_2_ composite	144	195.5	397	514	637	1356	1598.5	Doesn't indicated	—	51
TiO_2_ (anatase)	146	194	395	514	636	—	—	—	—	55

For the MWCNT coating, the values of each band (Fig. 3A) are similar to those shown in the literature, except for an extra Raman shift at 2942 cm^−1^ (second 2D (G′) band). The MWCNT coating exhibits three characteristic bands: disordered mode (D band), tangential mode (G band), and G′ band. The D mode, which appears for the MWCNTs coating at 1350 cm^−1^, represents the disorder in sp^2^-hybridized carbon atoms (graphene, which creates carbon nanotubes), the extent of sidewall defects or applied functionalization.^51^ The G band appears at 1588 cm ^−1^ and the G′ band, called the first overtone of the D band, at 2699 cm^−1^, which are called the two modes representing graphitic materials. The G mode is related to the stretching of the C–C bond, and the G′ mode can be distinguished for non-defect sp^2^ carbon materials.^51^ The second shift for the G′ band is found at 2942 cm^−1^, as observed several times for single-wall carbon nanotubes (SWCNTs) and double-wall carbon nanotubes (DWCNTs), and assigned to the tubes inside CNTs, whose diameter is smaller than the outer ones or resonance with the incident and scattered light or presence of structural defects, which might be eliminated after thermal treatment.^52^ The D and G band intensity (*I*_D_/*I*_G_) ratios are used to identify the degree of structural defects or the level of functionalization of MWCNTs.^51,53^ The lowest *I*_D_/*I*_G_ ratio occurs when more structural defects appear, which are the characteristics of the MWCNTs compared to those of SWCNTs and DWCNTs. The *I*_D_/*I*_G_ ratio for the MWCNT coating is about 1.34. The literature reports an *I*_D_/*I*_G_ ratio of 1.1437 (ref. 51) or 1.46 (ref. 54), according to our results.

The values of Raman shifts of the MWCNT/TiO_2_ coating (Fig. 3B) are close to those shown in the literature for the anatase phase of the titania, as illustrated in Table 4. Five optical phonon modes can be distinguished, and the assignment of the bands indicated is based on ref. 55 and 56. All of them represent five modes in the range of 150–630 cm^−1^ in accordance with Kamil *et al.* results,^51^ as shown in Table 4. Fig. 3B also demonstrates the mode characteristics of the MWCNT coating, which shifted and broadened and possessed a lower *I*_D_/*I*_G_ ratio of 0.97. It is evident that the addition of titania to the MWCNT coating resulted in more structural defects than the bare MWCNT coating. The shift of the D and G bands is due to the interaction between the TiO_2_ nanoparticles and the MWCNT coatings,^51,57^ with the *I*_D_/*I*_G_ ratio slightly higher than that shown by David *et al.* for the MWCNT/TiO_2_ film, which is 0.842.^57^ The archived values of the modes for both the MWCNTs and the MWCNT/TiO_2_ coating are slightly different from those reported in the literature, presumably owing to the effect of some impurities or a substrate.

### 3.3 Adhesion determination

**Fig. 4:**
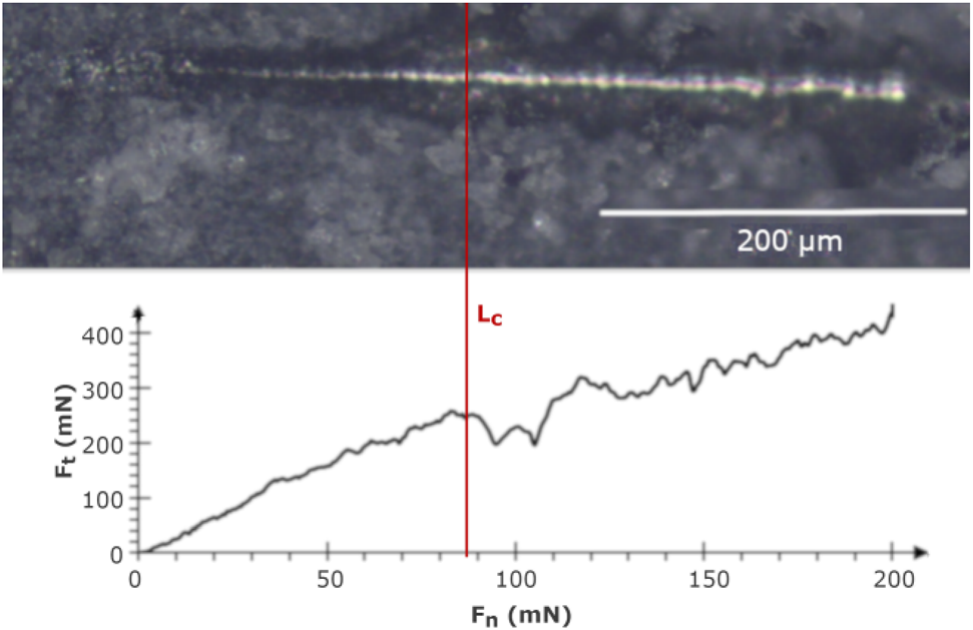
The nano-scratch test result for the MWCNT/TiO_2_ coating, with an indicated *L*_c_.

Fig. 4 shows the adhesion nano-scratch test result for the MWCNT/TiO_2_ coating, with the indicated *L*_c_ value demonstrating the moment of the coating delamination, where *F*_n_ is the normal force and *F*_t_ is the friction force.

Table 5 shows the estimated values of *L*_c_ and critical friction *F*_c_ for the examined coatings. The MWCNT/TiO_2_ coating demonstrates an almost 3.5-fold higher *L*_c_ value than the MWCNT coating, showing an improved adhesion strength. The adhesion strength measured by the shear strength test (according to ASTM standard F1044-99) for the MWCNT/TiO_2_/HAp coating demonstrated the adhesion strength for the coating EPD-deposited at 50 V for 1 min as 11.9 ± 3.3 MPa.^58^ The literature values of adhesion strength are then comparable to our results, within the limits of an experimental error. The adhesion can depend on mechanical properties, such as hardness, Young's modulus, and coating thickness, thus not only the coating composition determines the interface between a coating and a substrate. Therefore, the hardness of the tough TiO_2_ agglomerates on the surface of the MWCNT coating, together with its good fit to the substrate material, allows the coating to adhere better to the Ti13Nb13Zr substrate. The hardness of TiO_2_ can be taken as 1 GPa and that of the MWCNTs as 0.204 GPa.^59,60^ Owing to the higher hardness of TiO_2_ nanoparticles, the indenter tip encountered the titania particles on its way during the nano-scratch test and passed through the MWCNT/TiO_2_ coating at a higher *L*_c_ than in the MWCNT coating.

**Table 5:** The results of critical parameters after the nano-scratch test, where the result is presented as mean ± SD (*n* = 5)

Material	Critical load (*L*_c_) (mN)	Critical friction (*F*_c_) (mN)
MWCNTs	25.3 ± 1.9	41.2 ± 2.1
MWCNTs/TiO_2_	88.3 ± 1.8	248.5 ± 3.7

The adhesion strength also depends on coating thickness, coating structure, substrate architecture (evaluated using surface preparation), and method of synthesis.^63^ As regards the MWCNT/TiO_2_ coating thickness, it was assessed previously at 2.016 μm,^36^ while the thickness of the TiO_2_ coatings below 3 μm was reported to promote the loss of adhesion originating from the presence of titania agglomerates.^63^

There is one more parameter describing the adhesion strength of the coating, which is the ratio of hardness to reduced Young's modulus (*H*/*E*_r_), reported previously in ref. 36, which describes the coating endurance for substrate deflections under load. The results of the *H*/*E*_r_ ratio for the MWCNT and MWCNT/TiO_2_ coatings were 0.005 and 0.013, respectively,^36^ which agree with the present results of the nano-scratch test, demonstrating the positive effect of the use of titania.

### 3.4 Corrosion behavior

The corrosion resistance test's results are shown in Table 6. The MR and MWCNT corrosion resistance parameters have been discussed previously.^64^

**Table 6:** Corrosion test results for examined surfaces

Material	*E*_corr_ (V (SCE))	*j*_corr_ (nA cm^−2^)
MR^a^	−0.019 ± 0.007	38.66 ± 5.92
MWCNTs^a^	−0.109 ± 0.002	325.61 ± 33.61
MWCNT/TiO_2_	−0.206 ± 0.003	97.42 ± 9.39

a Results previously discussed in ref. 64.

The addition of TiO_2_ to MWCNT coatings lowers the *j*_corr_ and *E*_corr_ of the MWCNT coating, thus improving its corrosion resistance. The same result was reported for CNT/TiO_2_ coating deposited on other substrates, such as Mg–Zn–Ca alloy,^65^ MgZn alloy,^66^ and HA–Ti–MWCNTs on TiNi alloy.^37^ Compared to the MR material's corrosion parameters, the application of MWCNT/TiO_2_ coating weakens the corrosion resistance of the substrate Ti13Nb13Zr alloy. For composite material comprising MWCNTs and TiO_2_, excellent corrosion resistance was observed,^67^ but the MWCNT coating had a porous structure that enhanced the transport of aggressive ions into the substrate and localized corrosion. Moreover, adding TiO_2_ into the coating decreases its porosity by filling pores and voids, thus increasing the corrosion resistance of the MWCNT coating, as observed in other reports.^65,66^

### 3.5 Wettability

**Fig. 5:**
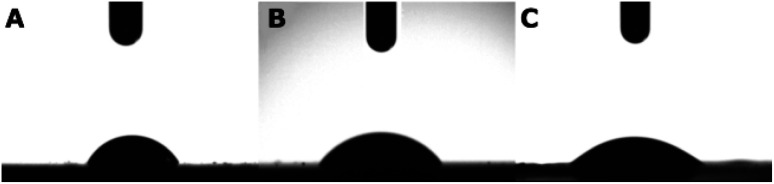
Results of water contact angle measurements for MR (A), the MWCNT coating (B), the MWCNT/TiO_2_ coating (C), where the results are presented as mean ± SD (*n* = 3).

Fig. 5 and Table 7 show the wettability measurement results for the examined surfaces. All of the surfaces are hydrophilic and both the MWCNTs and the MWCNT/TiO_2_ coatings demonstrate a contact angle between 50 and 60°, which is required for biomedical applications. The achieved results for the MWCNT coating are in accordance with our previous data shown in ref. 68. For the MWCNT/TiO_2_ materials, the literature demonstrates different results. For the MgZn/5TiO_2_–0.5MWCNT composite (composed of 5 wt% TiO_2_ and 0.5 wt% MWCNTs), the CA was reported as 87.0 ± 2.1°.^66^ Here, the difference might result in the effect of MgZn in the composite.

**Table 7:** Results of water contact angle measurements for the examined surfaces

Material	CA left (°)	CA right (°)	Mean CA (°)
MR	74.22 ± 4.96	73.09 ± 8.46	73.65 ± 6.58
MWCNTs	55.02 ± 1.02	55.84 ± 1.63	55.43 ± 1.29
MWCNTs/TiO_2_	57.16 ± 2.01	59.46 ± 1.29	58.31 ± 1.60

Ho *et al.* reported the CA for the MWCNT/TiO_2_ membrane of 45.55 ± 1.13°,^69^ which is slightly lower than the CA of our MWCNT/TiO_2_ coating.

### 3.6 Biological characterization

**Fig. 6:**
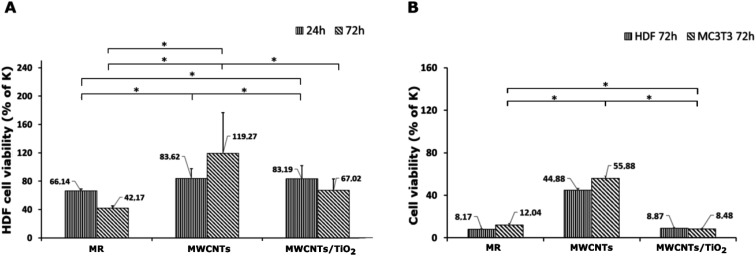
Viability of HDF cells after 24 and 72 h in direct test (A) and of HDF and MC3T3 cells after 72 h in indirect test (B), assayed with the MTT test (*n* = 3, * at a 0.05 level all the data was significantly drawn from a normally distributed population according to the Shapiro–Wilk normality test and according to one-way ANOVA the data are significantly different). The control values (100% cell viability) were assayed for untreated cells.

**Fig. 7:**
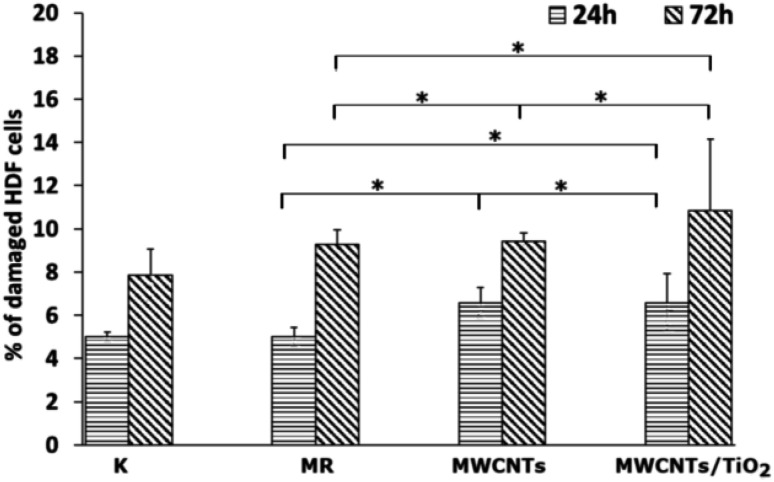
LDH activity in the culture media of HDF cells for 24 and 72 h (*n* = 3, * at a 0.05 level all the data was significantly drawn from a normally distributed population according to the Shapiro–Wilk normality test and according to one-way ANOVA the data are significantly different).

The results of the *in vitro* cytotoxicity studies are shown in Fig. 6 and 7. The viability of the HDF and MC3T3 cells was assayed using the MTT test. The highest cell viability for both HDF and MC3T3, and in both the direct and indirect tests, was observed for the MWCNT coating.

The MWCNT coating in the direct test demonstrated a slight decrease in HDF cell viability after 24 h, followed by an increase from 83.62% to 119.27% of the control after 72 h of incubation (Fig. 6A). It can be underpinned by the topography of the MWCNT coating, which promotes cell adhesion on a porous structure during the first 24 h; then, it also supports cell proliferation. There are reports on the carboxylated MWCNT–chitosan material's ability to achieve a porous microstructure, improving cell adhesion.^70^ However, the HDF cell viability of the MWCNT/TiO_2_ coating in the direct test after 24 h of incubation was negligibly lower than that for the MWCNT coating, while after 72 h, the HDF cell viability for the MWCNT/TiO_2_ coating considerably decreased to 67.02% of the control but was still higher than that of the pure substrate material. Herein, we can conclude that the topography of the MWCNT/TiO_2_ coating is as desirable as that of the MWCNT coating for cell adhesion, while after 72 h, the TiO_2_ nanoparticles started to have a stronger toxic effect on HDF cells and decreased their proliferation rate. According to the literature, the cytotoxicity of MWCNT/TiO_2_ films is dose-dependent, but there are also reports about the non-toxic effect of MWCNTs decorated with TiO_2_ after 24 h and 72 h, where the HDF cell viability was similar to that of the control, and the MWCNT_TiO_2_ composite concentrations were 0.02 and 0.05%.^57^ However, the strong toxic influence of crystal phase TiO_2_ and a decrease in A549 and MCF-7 cell viability have also been reported in the literature.^71^

The HDF and MC3T3 cell viability in an indirect test (Fig. 6B) was surprisingly low, suggesting that the tested specimens may have a toxic effect on the cells. In the case of the MWCNT coating, HDF cell viability in an indirect test is the highest and reaches 44.88% of the control but is still too low for successful application in biomedicine. The MC3T3 cells in the indirect test exhibited a viability of 55.88% of the control, but it is still unsatisfactory. These observations can be explained by toxic substances (*e.g.* some impurities) released from the material surface or with nutrients and growth factor depletion through their adsorption on the coating surface. Undoubtedly, the processes underlying this phenomenon deserve further elucidation. According to the literature, a carboxylated MWCNT–chitosan composite sol–gel material with osteogenic growth peptide (OGP(10–14)) showed potential for use in bone regeneration.^70^ Additionally, MWCNT scaffolds showed higher MC3T3 cell viability contrary to poly(lactic-*co*-glycolic) acid (PLGA),^72^ and the MWCNT layer on collagen-coated titanium plates supported cell proliferation.^73^ The anodized TiO_2_ nanoparticles (NPs) on titanium EPD coated with CNTs showed higher MC3T3 proliferation than TiO_2_ NPs,^74^ whereas a similar MWCNT coating reported by Park *et al.* demonstrated approximately 25% lower cell proliferation after 5 days than pure titanium.^75^

The LDH activity in the culture media (Fig. 7) was determined to check whether the MWCNT and MWCNT/TiO_2_ coatings (or substances released by them) have the ability to damage cell membranes and induce necrosis of HDF cells. After 24 h, both the MWCNT and the MWCNT/TiO_2_ coatings demonstrated a higher percentage of damaged HDF cells than the substrate material, but these values did not exceed 11% of the cells. However, after 72 h, the amount of damaged HDF cells was almost the same for the MWCNT coating (9.43%) and substrate material (9.29%), while the MWCNT/TiO_2_ coating achieved 10.86% of the damaged cells. The literature shows that LDH release from MWCNTs is dose- and time-dependent. The MWCNT dose of 40 μg mL^−1^ and the longer exposure time of HDF cells to carbon nanotubes induced cell death. However, we can see that the percentage of damaged cells exposed to MWCNTs is time dependent and much lower than that reported in the literature.^76,77^

## 4 Conclusions

In this study, employing the EPD method, we obtained a uniformly distributed MWCNT coating agglomerated with titania particles (of anatase structure, confirmed by Raman spectroscopy) deposited on the Ti13Nb13Zr substrate material to check its adhesion strength, corrosion resistance, wettability and biocompatibility with HDF and MC3T3 cells.

The adhesion strength of the MWCNT/TiO_2_ coating was 3.5-fold improved compared to that of the MWCNT coating owing to the tough and good fitting of titania to the substrate material, which was confirmed by calculating the *H*/*E*_r_ nanoindentation test parameter, reported previously in ref. 36.

The addition of titanium dioxide to the MWCNT coating resulted in more than 3-fold higher corrosion resistance than the basic MWCNT coating. TiO_2_ particles fill the inside of the MWCNT coating pores, making them denser. The MWCNT/TiO_2_ coating still exhibited worse corrosion resistance compared to the substrate material owing to its relatively high porosity, which is, however, the advantage of coatings intended for medical applications.

The results of biological tests confirmed that the improved mechanical and physicochemical properties of the surface, such as high porosity and wettability, in MWCNTs alone and MWCNT/TiO_2_-coated material support cell adhesion. The modified coatings may also release toxic substances into the culture medium, thus resulting in a decrease in proliferation.

The characterized coatings may be promising for biomedical applications, but they undoubtedly require further research and improvement, for example by reduction of the TiO_2_ content in the coating.

## Author contributions

D. Rogala-Wielgus: conceptualization, methodology, visualization, investigation, formal analysis, resources, writing – original draft, writing – review & editing; B. Majkowska-Marzec: conceptualization, visualization, project administration; A. Zieliński: conceptualization, investigation, supervision, writing – review & editing; K. Roszek: investigation, resources, validation; M. Liszewska: investigation, resources, validation. All authors have approved the final version of the manuscript.

## Conflicts of interest

There are no conflicts to declare.

## Supplementary Material
